# Just in time: detecting cardiac arrest with smartwatch technology

**DOI:** 10.1016/S2589-7500(24)00020-7

**Published:** 2024-03

**Authors:** Sulaiman Somani, Albert J Rogers

**Affiliations:** Department of Medicine and Cardiovascular Institute, Stanford University School of Medicine, Stanford, CA 94305, USA

Sudden cardiac death is the cause of nearly 50% of all cardiovascular deaths and is often precipitated by sudden cardiac arrest.^1^ When cardiac arrest occurs outside of the hospital setting, it has a 9·1% survival rate to hospital discharge in the USA.^1^ Because there is a nearly 18% decrease in survival for every minute of delay in defibrillation,^2^ early detection and response to sudden cardiac arrest can mean the difference between life and death. However, a detection technology that fits into daily life, and yet provides accurate and actionable information, is a crucial unmet clinical need.

In *The Lancet Digital Health*, Roos Edgar and colleagues^3^ report the development of an algorithm for sudden cardiac arrest detection from photoplethysmography and accelerometry data from a smartwatch. The algorithm achieves an impressive performance, with 98% sensitivity and 99·9% specificity for circulatory arrest detection in a prospective validation cohort. The study is highly innovative. First, although the use of smartwatch data for the detection of atrial arrhythmias is well studied in the literature,^4,5^ leveraging smartwatch data for the detection of sudden cardiac arrest has not been rigorously studied. Second, the authors of the study collected data from patients wearing a smartwatch during induced circulatory arrest during cardiovascular procedures such as transcatheter aortic valve intervention, defibrillator implantation, and ventricular tachycardia ablation, which provided a controlled setting for the development and evaluation of the algorithm. Because sudden cardiac death might be the first indication of cardiovascular disease in up to 50% of patients, the use of this data is a creative solution to the otherwise scarcity of available monitoring data for these events. Third, the algorithm identified by the authors is intuitive and deterministic, based on a low-peripheral systemic blood flow state. Therefore, it broadly addresses both types of circulatory arrests, ventricular arrhythmia and pulseless electrical activity, and does so with an algorithmic transparency that is often unsatisfactory with artificial intelligence models.

Integration of the technology presented in this study into practice might address the unmet need in sudden cardiac arrest. For patients with a high risk of sudden cardiac arrest but do not warrant an implanted defibrillator, this technology might provide an earlier initiation of the survival pathway using a smartwatch. Furthermore, the algorithm was able to detect pulseless electrical activity, which current defibrillators do not detect and might be the most common rhythm in unwitnessed sudden cardiac arrest.^6^ The authors have an established partnership with a local citizen rescuer network, which is encouraging for the next steps in implementing an end-to-end response system, to be studied in the subsequent phases of the DETECT programme. Proof of the effectiveness of this system might open the door for the use of other detection technologies that have been previously reported on to facilitate an early response, such as non-contact agonal breathing detection by smart speakers or fall detection surveillance cameras.^7^ Algorithm refinement using larger datasets and multimodal sensors might improve detection performance and even permit the upstream prediction of arrests.^8^

As with any innovative study, there are several areas that provide room for future work. The patients in this study were evaluated in a controlled setting, with study personnel assisting in application of the smartwatch. When implemented for out-of-hospital arrest, there will be greater signal heterogeneity because of variation in device fitting, skin tones, disease phenotypes, and body positioning. The event rate for cardiac arrest in the training set was higher than in real-world settings, where these events are much rarer, and a false positive alarm rate of 0·09 (nine alarms per 100 h), or 2·16 alarms per day (even under a controlled setting), could become a burden to the wearer or response network. Careful calibration will be needed to minimise the risk of excessive false positives and the potentially quality-of-life issues from psychological distress or inappropriate response activations. Finally, care should be taken in finding an optimal integration point with response networks, taking all stakeholders into account. These limitations serve not to temper the excitement from this study but show the pathway for future work for its investigators.

To our knowledge, this is the first study to systematically investigate the detection of sudden cardiac arrest from smartwatch data, which represents a preliminary but solid foundation for the evolution of wearable technology in sudden cardiac arrest. We eagerly await subsequent work by the authors that might address the remaining barriers and accelerate the chain of survival in sudden cardiac arrest.
